# CLRM-11. BENCH TO BEDSIDE NEURO-ONCOLOGY: ADVOCATING FOR A CLINICALLY RELEVANT STRATEGY AS UNDERSCORED BY THE PANDEMIC

**DOI:** 10.1093/noajnl/vdab112.010

**Published:** 2021-09-21

**Authors:** Timothy Brown, Alireza Mansouri, Samer Zammar, Michael Glantz

**Affiliations:** 1 University of Pennsylvania, Philadelphia, PA, USA; 2 Penn State Health Milton S Hershey Medical Center, Hershey, PA, USA

## Abstract

**INTRODUCTION:**

The basic science research endeavor has been abundantly and astonishingly successful in the last three decades in elucidating the mechanisms of neuro-oncologic disease and in suggesting therapeutic strategies. Clinical successes have lagged behind, and translation of promising laboratory findings into clinical practice is rare. We hypothesize that one important reason for this discordance is the use of different paradigms for designing laboratory and clinical trials, and that utilizing clinically relevant procedures could improve laboratory study impact.

**METHODS:**

We identified all pre-clinical neuro-oncology therapeutic trials published in four high-impact journals between 11/2018 and 4/2019 and assigned a level of evidence (LOE) to each study using the American Academy of Neurology evidence classification system. We then identified all phase III trials of therapeutics for COVID and performed the same analysis on all preclinical studies preceding the trials.

**RESULTS:**

Of the 26 neuro-oncology articles identified, 85% had a LOE of IV and 15% were class III. An analysis of successful human trials showed significantly more high quality laboratory studies supporting “successful” compared to “unsuccessful” trials (p=0.048). This same pattern was identified in phase III trials of COVID. Twenty antiviral studies failed to meet the primary endpoint; all were preceded by class III or IV LOE preclinical studies. Eight evaluable phase three studies of COVID vaccines were identified, all of which met their primary endpoints. These were supported with a mix of Class I/II (n=4) and III/IV (n=4) preclinical studies. Higher LOE by AAN criteria is associated with successful COVID therapeutic trials (p=0.0034).

**CONCLUSIONS:**

Despite rigorous, elegant, and enlightening laboratory experiments, successful translation to human therapeutics remains rare. Envisioning basic science research through the lens of clinical therapeutics represents a challenging but surmountable paradigm shift that may reverse this pattern and create a more successful research enterprise in neuro-oncology and beyond.

